# Abnormal whole-brain voxelwise structure-function coupling and its association with cognitive dysfunction in patients with different cerebral small vessel disease burdens

**DOI:** 10.3389/fnagi.2023.1148738

**Published:** 2023-06-30

**Authors:** Xinyue Zhang, Changhu Liang, Na Wang, Yuanyuan Wang, Yian Gao, Chaofan Sui, Haotian Xin, Mengmeng Feng, Lingfei Guo, Hongwei Wen

**Affiliations:** ^1^Key Laboratory of Endocrine Glucose and Lipids Metabolism and Brain Aging, Ministry of Education, Department of Radiology, Shandong Provincial Hospital Affiliated to Shandong First Medical University, Jinan, Shandong, China; ^2^Department of Medical Imaging, Binzhou Medical University, Yantai, Shandong, China; ^3^Department of Radiology, Shandong Provincial Hospital, Cheeloo College of Medicine, Shandong University, Jinan, Shandong, China; ^4^Key Laboratory of Cognition and Personality (Ministry of Education), Faculty of Psychology, Southwest University, Chongqing, China

**Keywords:** cerebral small vessel disease, low frequency fluctuations, voxel-based morphometry, structure-function coupling, cognitive dysfunction, burden

## Abstract

Cerebral small vessel disease (CSVD) is a universal neurological disorder in older adults that occurs in connection with cognitive dysfunction and is a chief risk factor for dementia and stroke. While whole-brain voxelwise structural and functional abnormalities in CSVD have been heavily explored, the degree of structure-function coupling abnormality possible in patients with different CSVD burdens remains largely unknown. This study included 53 patients with severe CSVD burden (CSVD-s), 108 patients with mild CSVD burden (CSVD-m) and 76 healthy controls. A voxelwise coupling metric of low frequency fluctuations (ALFF) and voxel-based morphometry (VBM) was used to research the important differences in whole-brain structure-function coupling among groups. The correlations between ALFF/VBM decoupling and cognitive parameters in CSVD patients were then investigated. We found that compared with healthy controls, CSVD-s patients presented notably decreased ALFF/VBM coupling in the bilateral caudate nuclei and increased coupling in the right inferior temporal gyrus (ITG). In addition, compared with the CSVD-m group, the CSVD-s group demonstrated significantly decreased coupling in the bilateral caudate nuclei, right putamen and inferior frontal gyrus (IFG) and increased coupling in the left middle frontal gyrus and medial superior frontal gyrus. Notably, the ALFF/VBM decoupling values in the caudate, IFG and ITG not only showed significant correlations with attention and executive functions in CSVD patients but also prominently distinguished CSVD-s patients from CSVD-m patients and healthy controls in receiver operating characteristic curve research. Our discoveries demonstrated that decreased ALFF/VBM coupling in the basal ganglia and increased coupling in the frontotemporal lobes were connected with more severe burden and worse cognitive decline in CSVD patients. ALFF/VBM coupling might serve as a novel effective neuroimaging biomarker of CSVD burden and provide new insights into the pathophysiological mechanisms of the clinical development of CSVD.

## 1. Introduction

Cerebral small vessel disease (CSVD) represents a cluster of vascular diseases involving a variety of cerebral vessels, including arterioles, venules, and capillaries; it is believed to be a major factor in dementia, cognitive decline, mood disorders, gait impairment and stroke (Litak et al., 2020). Common imaging markers of CSVD include cerebral microbleeds (CMBs), white matter magnetic resonance hyperintensities (WMHs) of presumed vascular origin, lacunes of presumed vascular origin, enlarged perivascular spaces (PVSs), brain atrophy and recent small subcortical infarcts (Wardlaw et al., 2013). Previous studies have proposed combining the above imaging markers to score the severity of CSVD, namely, the burden of CSVD, which can be used to predict the risk of dementia (Amin Al Olama et al., 2020) and is related to cognition and blood pressure in lacunar stroke patients (Staals et al., 2014). It has been shown that higher total CSVD burden was associated with overall cognitive ability in middle-aged and older Chinese people (Li X. et al., 2021) and can be a long-term strong predictor of cognitive decline and dysfunction (Jokinen et al., 2020). CSVD burden provides a better estimate of the overall effect of CSVD on the brain than a single imaging marker. Furthermore, previous studies have shown that the concept of a total score for small vessel disease in cerebral amyloid angiopathy is valid and can be used in prospective clinical studies (Charidimou et al., 2016). Therefore, stratification based on burden risk for other clinical studies of CSVD is reliable. However, in only a few studies have participants been grouped according to CSVD severity scores.

Currently, multimodal magnetic resonance imaging (MRI) techniques are widely applied to reveal the functional and structural alterations underlying CSVD pathology. For functional changes in CSVD, based on resting-state functional MRI (rs-fMRI), the amplitude of low frequency fluctuations (ALFF) metric, which can reliably and reproducibly reflect the level of regional functional neural activity (Yu-Feng et al., 2007), was used to reveal that CSVD patients with CMBs demonstrated higher ALFF values in the putamen, left precuneus and right insula (Feng et al., 2021). In addition, substantially increased fractional ALFF in the right inferior frontal gyrus and the left caudate were revealed in CSVD patients with gait disturbances (Zhou et al., 2020). Regarding structural changes in CSVD, voxel-based morphometry (VBM) research was used to reveal that CSVD patients showed substantially decreased gray matter (GM) and white matter (WM) volumes in the right inferior frontal gyrus and left medial superior frontal gyrus (Li et al., 2022). Furthermore, CSVD patients frequently experience brain functional changes accompanied by structural abnormalities (Telgte et al., 2018). At present, few studies have coupled ALFF and VBM to analyze neurological and psychiatric diseases, and it is a relatively blank field of current research to use coupling indicators to analyze CSVD. Some scholars (Li H. et al., 2021) combined ALFF and VBM analysis to find that patients with vascular mild cognitive impairment presented GM atrophy in the right precentral gyrus and inferior temporal gyrus and decreased ALFF in structures of the default mode network (DMN), which may be a calculable biomarker of cognitive impairment. Since the impacted brain areas are not consistently revealed by structural and functional MRI studies, it is more beneficial to directly study the association between structural and functional alterations in CSVD patients, which may help to elucidate the underlying neurobiological mechanisms of CSVD.

Emerging evidence indicates that the coupling of multimodal structural and functional imaging can be used to analyze a variety of neuropsychiatric diseases as it demonstrates additional messages and promotes the sensitivity of diagnosis compared to a single imaging modality (Wen et al., 2017). The present study showed that structural-functional deficits have been observed in patients with social anxiety disorder (Zhang et al., 2022), and the integration of structural and functional MRI contributes to identifying mild cognitive impairment (MCI), improving its diagnostic efficacy (Kim and Lee, 2013). Among the current numerous multimodal fusion analysis methods, structure-function coupling is a novel measure that describes the structural support for functional interaction, and increased structure–function coupling in the frontal association cortex may support functional specialization in connection with cognition (Baum et al., 2019). Importantly, previous studies (Zhao et al., 2015; Li H. et al., 2021) revealed that GM volume (GMV) alterations obtained by VBM analysis are highly related to ALFF deficits in CSVD patients with cognitive impairment. Therefore, ALFF/VBM coupling may be a reliable structure-function coupling metric that can reflect the neural mechanism underlying CSVD with cognitive impairment. Notably, Kang et al. (2022) recently developed a voxelwise coupling analysis of rs-fMRI and VBM parameters to assess structure–function decoupling and its importance in evaluating patients with nasopharyngeal carcinoma (NPC). They found that after radiotherapy, NPC patients showed increased rs-fMRI/VBM coupling parameters in the putamen, thalamus, and substantia nigra (SN) and decreased coupling parameters mainly in the medial temporal and prefrontal gyrus. However, despite these advances, structure-function decoupling in CSVD patients, especially those with different CSVD burdens, has not been explored.

In this study, we aimed to first compute ALFF and VBM maps and then establish an ALFF/VBM coupling metric to assess structure–function decoupling in CSVD patients with different burdens. Notably, in VBM research, the brain can be segmented into gray matter, white matter and cerebrospinal fluid (Nyatega et al., 2022). Therefore, ALFF can be coupled with WMV and GMV to explore alterations in different brain regions. Furthermore, we analyzed the correlation between ALFF/VBM coupling metrics and cognitive parameters in CSVD patients. Finally, it was hypothesized that ALFF/VBM would be a reliable and valid neuroimaging parameter that indicated structure–function decoupling under different CSVD burdens and that structure–function decoupling may be distinctly related to cognitive impairment.

## 2. Materials and methods

### 2.1. Participants

This cross-sectional study was approved for implementation by Shandong Provincial Hospital Affiliated with Shandong First Medical University’s institutional review board. Between December 2018 and January 2022, 53 patients with severe CSVD burden (CSVD-s age: 63.90 ± 6.23 years; 19 females) and 108 patients with mild CSVD burden (CSVD-m; age: 61.45 ± 7.79 years; 51 females) were recruited. We also included 76 age-, sex- and education-matched healthy controls (age: 60.55 ± 9.29 years; 42 females) in our study. All participants voluntarily signed an informed consent form before the research began.

According to current MRI consensus standards (Wardlaw et al., 2013), the criteria for inclusion were as follows: CMBs, WMHs of presumed vascular origin, lacunes of presumed vascular origin, enlarged PVSs, brain atrophy and recent small subcortical infarcts. The “total SVD burden” (Klarenbeek et al., 2013) was used to evaluate the seriousness of the CSVD, and a pragmatic ordinal scale of 0–4 was applied, reflecting the four mentioned MRI symbols of CSVD. One point was given if ≥1 CMB was observed; one point if an early syncretic deep WMH [Fazekas score (Fazekas et al., 1987) 2 or 3] or anomalous periventricular WMH progressing to the deep white matter (Fazekas score 3) was observed; one point if ≥1 lacune was observed; and one point if moderate to severe (grade 2–3) enlargement of a PVS in the basal ganglia was observed. Participants with scores of 0–1 were classified in the CSVD-m group, and those with scores of 2–4 were classified in the CSVD-s group.

The following conditions were criteria for exclusion: (1) a medical history of stroke, epilepsy, brain trauma, or tumor; (2) a medical history of alcohol or drug abuse; (3) a medical history of thrombolysis; (4) an occurrence of liver, heart, or kidney injury; (5) severe hypertension or acute complications of type 2 diabetes mellitus; and (6) a medical history of serious psychiatric or neurological illnesses.

### 2.2. MRI obtainment

A 3.0-Tesla MR system (Siemens Healthcare, Erlangen, Germany) equipped with a 32-channel head coil for signal reception was used to acquire MRI images. High-resolution 3D T1-weighted structural images were obtained by a magnetization-prepared rapid gradient echo (MPRAGE) sequence on the basis of the following settings: repetition time (TR)/echo time (TE) = 7.3/2.4 ms; flip angle = 9°; field of view (FOV) = 240 mm^2^ × 240 mm^2^; matrix size = 256 × 256; slice thickness = 1 mm; 192 slices; and slice thickness = 0.9 mm with no gap. The rs-fMRI images were obtained using a gradient echo-echo-planar imaging (GE-EPI) sequence with the following index: TR/TE = 1,500/30 ms; FOV = 240 mm^2^ × 240 mm^2^; matrix size = 64 × 64; slice thickness = 3 mm; slice gap = 1 mm; 50 axial slices; 200 volumes and 50 axial slices.

### 2.3. Cognitive assessments

The Montreal Cognitive Assessment (MoCA) Beijing version^1^ was given to all participants (Bergeron et al., 2017). The optimal threshold for identifying cognitive dysfunction was 13/14 points for illiteracy, 19/20 for those with 1–6 years of schooling and 24/25 for those with 7 or more years of schooling (Lu et al., 2011). Additionally, other executive abilities were tested, including adaptability, working memory, and inhibiting ability. In brief, these tests included the symbol digit modalities test (SDMT) to gauge information processing speed and attention (Benedict et al., 2017), the trail-making test (TMT), which measures information processing speed, visual search, attention and motor coordination (Wei et al., 2018), the Rey auditory verbal learning test (AVLT) to evaluate verbal memory skills (Putcha et al., 2019), and the Stroop color-word test (SCWT) (Scarpina and Tagini, 2017). The test administrator had professional training and appropriate credentials and knew nothing about the subject grouping in advance.

### 2.4. Data preprocessing and function-structure coupling analysis

We adopted the standard procedure for the preprocessing of rs-fMRI data following our previous studies (Wen et al., 2018; Feng et al., 2021) and used the Data Processing and Analysis for Brain Imaging toolbox (DPABI v6.0^2^). Briefly, the main procedures were as follows: (1) the removal of the first ten time points; (2) slice timing correction; (3) head motion correction, for which the exclusion criteria were a maximum head motion of 3.0 degree and 3.0 mm, with mean framewise displacement (FD) >0.2 mm (Jenkinson et al., 2002); (4) the regression of nuisance covariates, which included signals from WM and cerebrospinal fluid (CSF), and 24 rigid body motion parameters; (5) normalization to the MNI standard space at 3 mm isotropic voxel resolution by DARTEL (Ashburner, 2007); (6) spatial smoothing with an isotropic Gaussian kernel of 4 mm full width at half maximum (FWHM); and (7) the calculation of ALFF by transforming the smoothed time series of each voxel into frequency domain data by a fast Fourier transform (FFT). The square root of the power spectrum was calculated and averaged over a predetermined frequency range (0.01–0.08 Hz) at each voxel (Yu-Feng et al., 2007). Finally, the ALFF per voxel was separated by the global ALFF (mean ALFF of all voxels) to generate the standardized ALFF metric for further coupling analysis.

The T1W images were preprocessed and analyzed using VBM-DARTEL analysis as described in our recent studies (Wen et al., 2017; Li et al., 2022) based on the SPM8^3^ toolbox. Briefly, every T1W image was manually readjusted by placing the origin at the anterior commissure. Afterward, using SPM’s New Segment tool, the aligned images were separated into GM, WM, and CSF in the original space using unitive segmentation. Afterward, to create a variety of aligned GM and WM images, all of the segmented GM and WM images were highly modified. The DARTEL algorithm was used to construct the study-specific GM templates, and every aligned GM image was then warped to the template, producing numerous flow fields that specified distortion. The GM/WM images were modified to correct volume alterations after being spatially adjusted to the MNI standard space. GMV and WM volume (WMV) maps were smoothed with a Gaussian kernel of 4 mm FWHM.

Subsequently, we combined rs-fMRI and VBM metrics for structure-function coupling analysis. Since both the ALFF maps and the regulated VBM maps (GMV/WMV) for each subject were normalized to the same MNI standard space, based on a previous study (Kang et al., 2022), voxelwise ALFF/VBM coupling research was executed by determining the ratio of the ALFF value to the GMV/WMV value per voxel at the same coordinate.

### 2.5. Between-group statistical comparisons and correlation analysis

One-way analysis of variance (ANOVA) and least significant difference (LSD) post-hoc tests were applied to assess differences in age, education, head motion and cognitive parameters among the three groups, and a chi-square test was performed to probe the difference in sex.

For the voxelwise ALFF/VBM coupling metric, one-way analysis of covariance (ANCOVA) was used to assess differences among the three groups while controlling age, sex, education and head motion as covariates. Then, LSD post-hoc tests were used, and the corrected *p*-values for comparison between each pair of groups were calculated. After the corrected *p*-value maps were converted to *z* value maps, Gaussian random field (GRF) correction (voxel level *p* < 0.001, cluster level *p* < 0.05) was performed on the *z* value maps using DPABI utilities (Yan et al., 2016; Bansal and Peterson, 2018). The peak voxel coordinates of clusters were expounded in MNI space, and the AAL atlas (Tzourio-Mazoyer et al., 2002) was used for anatomical definition. We further extracted the mean ALFF/VBM coupling values from the identified altered clusters and calculated the Pearson correlation coefficients between the coupling metrics and cognitive test scores for the CSVD-s and CSVD-n groups using SPSS v24.0 software.

### 2.6. Receiver operating characteristic curve analysis

The results of our recent studies (Feng et al., 2021; Li et al., 2022) suggest that ALFF and VBM changes may be reliable imaging metrics to discriminate CSVD patients from healthy controls. The mean ALFF/VBM coupling values of markedly altered clusters in ANCOVA were extracted for receiver operating characteristic (ROC) curve analysis using MedCalc Statistical Software^4^ to confirm the performance of these biomarkers for discriminating CSVD patients from healthy controls. We calculated the greatest Youden index (specificity + sensitivity–1) (Fluss et al., 2005), as well as the corresponding specificity, sensitivity, and 95% confidence intervals (CIs) for all clusters, to provide a summary of the tests’ overall diagnostic efficacy.

## 3. Results

### 3.1. Demographic and cognitive parameters of the participants

The demographic and cognitive parameters in every group are summarized in Table 1. The CSVD-s group exhibited obviously decreased SDMT, AVLT, and MoCA scores and distinctly increased TMT and SCWT scores compared with the CSVD-m and control groups, apart from there being no visible distinction in MoCA scores between the CSVD-s and CSVD-m groups. In addition, the patients in the CSVD-m group showed distinctly lower SDMT scores and higher SCWT scores than those in the control group. No apparent differences were found in age, sex, education or head motion (mean FD) among groups.

**TABLE 1:** Demographic and clinical characteristics of CSVD patients and healthy controls.

Characteristic	CSVD-s	CSVD-m	HC	*P*-value (ANOVA/χ ^2^)	*P*-value (*post-hoc*)		
					CSVD-s vs. HC	CSVD-s vs. CSVD-m	CSVD-m vs. HC
**Gender**	34 M/19 F	57 M/51 F	34 M/42 F	0.094χ^2^	–	–	–
**Age (y)**	63.90 ± 6.23	61.45 ± 7.79	60.55 ± 9.29	0.060^a^	–	–	–
**Education (y)**	11.26 ± 3.22	12.07 ± 3.14	12.67 ± 3.35	0.053^a^	–	–	–
**MoCA**	24.47 ± 2.91	25.38 ± 3.57	26.39 ± 3.57	0.009^a^	0.002	0.121	0.052
**AVLT**	54.66 ± 13.5	61.05 ± 12.19	64.6 ± 11.94	<0.001^a^	<0.001	0.003	0.059
**SDMT**	26.56 ± 11.94	32.16 ± 12.23	40.57 ± 13.53	<0.001^a^	<0.001	0.010	<0.001
**SCWT**	178.68 ± 59.41	145.47 ± 44.98	131.78 ± 31.08	<0.001^a^	<0.001	<0.001	0.045
**TMT (B-A)**	161.8 ± 91.09	125.59 ± 106.34	104.84 ± 80.08	0.005^a^	0.001	0.028	0.152
**FD_Jenkinson**	0.13 ± 0.04	0.12 ± 0.04	0.11 ± 0.04	0.135^a^	–	–	–
**Lacunes**	22 (41.5%)	2 (1.9%)	–	<0.001χ^2^			
**WMHs**	52 (98.1%)	105 (97.2%)	–	<0.001χ^2^			
**PVSs**	47 (88.7%)	34 (31.5%)	–	<0.001χ^2^			
**CMBs**	34 (64.2%)	13 (12.0%)	–	<0.001χ^2^			

χ^2^: chi-square test.

^a^ANOVA test. MoCA, montreal cognitive assessment; AVLT, sum of Rey auditory verbal learning test (N1-7); SDMT, symbol digit modalities test; SCWT, sum of Stroop color-word test (stroop1–3); TMT, the trail-making test; TMT (B-A), the difference between TMT-B and TMT-A; CSVD-s, severe CSVD burden (score ≥2) group; CSVD-m, mild CSVD burden (score ≤1) group; HC, healthy controls; FD Jenkinson, framewise displacement (Jenkinson et al., 2002); Lacunes, one of the imaging markers of CSVD; WMHs, white matter magnetic resonance hyperintensities; PVSs, enlarged perivascular spaces; CMBs, cerebral microbleeds.

### 3.2. Abnormal ALFF/VBM coupling among groups

**FIGURE 1:**
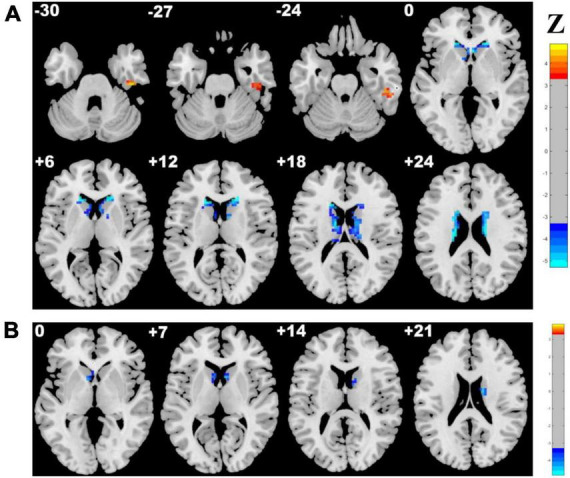
Brain clusters revealing distinctly abnormal ALFF/VBM coupling in the CSVD-s group compared with the control group. Clusters with significantly changed **(A)** ALFF/GMV and **(B)** ALFF/WMV coupling identified by ANCOVA and LSD *post-hoc* test with GRF correction (voxel level *p* < 0.001, cluster level *p* < 0.05). The red–yellow and blue–light blue color bars represent the level of significance for ALFF/VBM coupling increases and decreases in the CSVD-s group, respectively.

**FIGURE 2:**
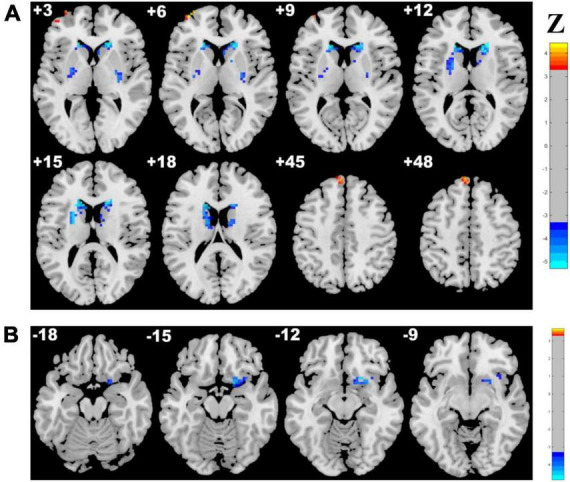
Brain clusters revealing distinctly abnormal ALFF/VBM coupling in the CSVD-s group compared with the CSVD-m group. Clusters with significantly changed **(A)** ALFF/GMV and **(B)** ALFF/WMV coupling are shown in red–yellow and blue–light blue colors, representing the level of significance for ALFF/VBM coupling increases and decreases in the CSVD-s group, respectively.

Adopting the voxelwise structure-function coupling metric, we found multiple disrupted regions among the three groups. Specifically, compared with patients in the control group, the patients in the CSVD-s group showed significantly decreased ALFF/GMV and ALFF/WMV coupling values in the bilateral caudate clusters and increased ALFF/GMV coupling in the right inferior temporal gyrus (Figure 1 and Table 2). In addition, compared with patients in the CSVD-m group, patients in the CSVD-s group revealed obviously decreased ALFF/GMV coupling in the bilateral caudate and right putamen clusters, decreased ALFF/WMV coupling in the right inferior frontal gyrus (IFG) and increased ALFF/GMV coupling in the left middle frontal gyrus (MFG) and medial superior frontal gyrus (SFGmed) (Figure 2 and Table 3). No significant ALFF/VBM coupling distinction was found between the CSVD-m and control groups.

**TABLE 2:** Clusters with significantly altered ALFF/VBM coupling in the CSVD-s group compared with the control group.

Metrics and condition	Brain regions	Cluster size	z score of peak voxel	MNI coordinates of peak voxel		
				x	y	z
ALFF/GMV CSVD-s < control	Right caudate	173	5.295	15	27	0
	Left caudate	159	5.295	−18	27	6
ALFF/GMV CSVD-s > control	Right inferior temporal gyrus	31	4.901	45	−21	−30
ALFF/WMV CSVD-s < control	Right caudate	39	4.586	15	−6	21
	Left caudate	25	4.271	−9	9	0

ANCOVA and LSD *post-hoc* tests were applied to identify the altered ALFF/VBM coupling among groups with Gaussian random field (GRF) multiple comparison corrections (voxel level *p* < 0.001, cluster level *p* < 0.05). CSVD-s, severe CSVD burden (score ≥2) group.

**TABLE 3:** Clusters with significantly altered ALFF/VBM coupling in the CSVD-s group compared with the CSVD-m group.

Metrics and condition	Brain regions	Cluster size	z score of peak voxel	MNI coordinates of peak voxel		
				x	y	z
ALFF/GMV CSVD-s < CSVD-m	Left caudate	181	5.295	−18	27	6
	Right caudate	137	5.295	15	21	15
	Right putamen	23	4.360	27	−9	3
ALFF/GMV CSVD-s > CSVD-m	Left middle frontal gyrus	11	4.388	−36	60	6
	Left medial superior frontal gyrus	14	4.046	−6	51	48
ALFF/WMV CSVD-s < CSVD-m	Right inferior frontal gyrus	42	4.427	18	9	−15

ANCOVA and LSD *post-hoc* tests were applied to identify the altered ALFF/VBM coupling among groups with Gaussian random field (GRF) multiple comparison corrections (voxel level *p* < 0.001, cluster level *p* < 0.05). CSVD-s, severe CSVD burden (score ≥2) group; CSVD-m, mild CSVD burden (score ≤1) group.

### 3.3. ROC curve analysis results

**FIGURE 3:**
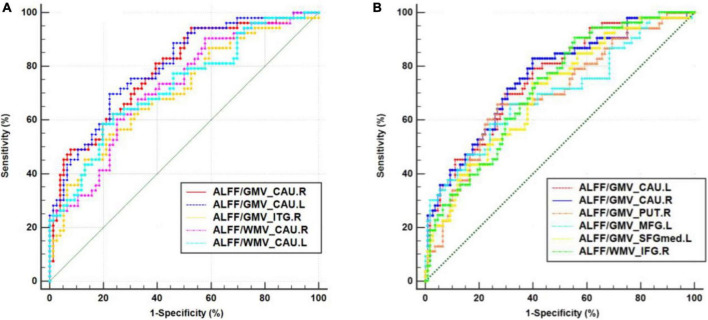
Receiver operating characteristic (ROC) curve analysis results. ROC curves for brain clusters that discriminate **(A)** CSVD-s patients from healthy controls and discriminate. **(B)** CSVD-s patients from CSVD-m patients. CAU, caudate; ITG, inferior temporal gyrus; MFG, middle frontal gyrus; SFGmed, medial superior frontal gyrus; IFG, inferior frontal gyrus.

For discriminating CSVD-s patients from healthy controls, the mean ALFF/GMV coupling value of the left caudate achieved the best performance, and for discriminating CSVD-s patients from CSVD-m patients, the mean ALFF/GMV coupling value of the right caudate achieved the best performance in view of the Youden index, region under the ROC curve (AUC) and 95% CIs. Each changed brain cluster met the *p* < 0.001 AUC significance threshold, indicating that the discoveries are meaningful and that these parameters have potential as diagnostic neuroimaging biomarkers (Figure 3 and Table 4).

**TABLE 4:** The statistics of ROC curve analysis for altered brain clusters that discriminate CSVD-c patients from healthy controls.

Clusters	SEN	SPE	AUC	95% CIs
**Task: Distinguish between CSVD-s and control groups**				
ALFF/GMV_Right caudate	94.34%	47.37%	0.786*	0.705–0.853
ALFF/GMV_Left caudate	69.81%	77.63%	0.799*	0.720–0.865
ALFF/GMV_Right ITG	54.72%	77.63%	0.707*	0.620–0.784
ALFF/WMV_Right caudate	60.38%	75.00%	0.722*	0.636–0.797
ALFF/WMV_Left caudate	58.49%	80.26%	0.723*	0.638–0.799
**Task: Distinguish between CSVD-s and CSVD-m groups**				
ALFF/GMV_Left caudate	69.81%	70.37%	0.757*	0.683–0.821
ALFF/GMV_Right caudate	83.02%	60.19%	0.759*	0.685–0.823
ALFF/GMV_Right putamen	66.04%	73.15%	0.698*	0.621–0.768
ALFF/GMV_Left MFG	66.04%	68.52%	0.696*	0.619–0.766
ALFF/GMV_Left SFGmed	73.58%	60.19%	0.702*	0.625–0.771
ALFF/WMV_Right IFG	90.57%	44.44%	0.719*	0.643–0.787

SEN/SPE, sensitivity/specificity corresponding to maximum Youden index; AUC, area under the ROC curve; *p < 0.001; CIs, confidence intervals; ITG, inferior temporal gyrus; MFG, middle frontal gyrus; SFGmed, medial superior frontal gyrus; IFG, inferior frontal gyrus.

### 3.4. Associations between ALFF/VBM coupling and cognitive parameters

**FIGURE 4:**
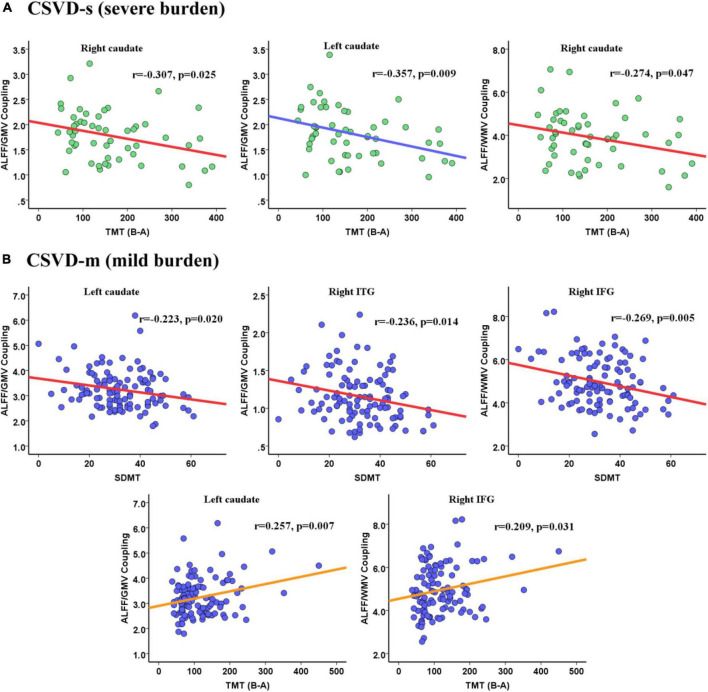
Pearson correlations between the ALFF/VBM coupling values and cognitive scores for the **(A)** CSVD-s group and **(B)** CSVD-m group.

Pearson’s correlation analysis was performed to estimate the relevance between the mean coupling values of aberrant clusters and cognitive scores in CSVD-s and CSVD-m patients. In CSVD-s patients, the mean ALFF/GMV value of the bilateral caudate (*r* = −0.307, *p* = 0.025 and *r* = −0.357, *p* = 0.009) and the mean ALFF/WMV value of the right caudate (*r* = −0.274, *p* = 0.047) showed significant negative correlations with TMT scores (Figure 4A). In CSVD-m patients, the mean ALFF/GMV value of the left caudate (*r* = −0.223, *p* = 0.020) and right ITG (*r* = −0.236, *p* = 0.014) and the mean ALFF/WMV value of the right IFG (*r* = −0.269, *p* = 0.005) showed obvious negative correlations with SDMT scores, while the mean ALFF/GMV value of the left caudate (*r* = 0.257, *p* = 0.007) and the mean ALFF/WMV value of the right IFG (*r* = 0.209, *p* = 0.031) showed evident positive correlations with TMT scores (Figure 4B).

## 4. Discussion

As far as we know, this study designed to detect structure-function decoupling in patients with different CSVD burdens by coupling analysis of rs-fMRI and VBM metrics is novel. In our research, an ALFF/VBM metric was applied to reflect the coupling between spontaneous brain activity and GM/WM volume. For each participant, the voxelwise ALFF/VBM ratio characterizes the quantity of regional neuronal activity requirement per unit of regional GM/WM morphological alterations, which could measure the structure–function coupling for a concrete region or voxel. Compared with the patients in the CSVD-m and control groups, ALFF/VBM coupling was significantly altered in CSVD-s patients and was prominently associated with TMT scores. Specifically, decreased ALFF/GMV and ALFF/WMV coupling values in the CSVD-s group were observed mainly in the basal ganglia and right IFG, and increased coupling mainly occurred in the frontal and temporal cortical areas. The increase and decrease of coupling values in different regions are both involved in the structural and functional decoupling, which indicated that the structure and function of the brain in CSVD patients were dysregulated; that is, the duration and intensity of the activity were altered along with the structural damage of corresponding brain regions. Overall, our findings highlighted the importance of multimodal MRI fusion and provided insights promoting a better comprehension of the correlation between structure–function decoupling and cognitive deficits in patients with different CSVD burdens.

Notably, the disturbed basal ganglia regions mainly contain the caudate and putamen. The caudate and putamen make up the dorsal striatum, which is the pathway to the basal ganglia. Furthermore, the plasticity of the striatum may be a crucial neuronal matrix for motor control and procedural memory (Kreitzer and Malenka, 2008). Thus, gait disturbance may result from decreased structure-function coupling in this area. Recent research has demonstrated that CSVD-induced microscopic brain structural changes impact motor ability in the upper and lower limbs (Su et al., 2017), and slower gait speed is related to weaker basal ganglia connectivity in the resting state (Karim et al., 2020), supporting this viewpoint. Furthermore, gait abnormalities predict declines in overall cognitive, memory, and language abilities (Blumen et al., 2023). On the other hand, decoupling also occurs in the temporal and frontal lobes. It was shown that cognitive decline associated with cerebrovascular disease (including CSVD) affects frontal executive function (Dichgans and Leys, 2017), particularly episodic memory. The gradual decline in cognitive function has been related to frontal and parietal hypometabolism and inferior temporal cortical thinning (Halawa et al., 2019). The frontal lobes play a prominent role in human cognitive functions and socioemotional function, and several neurodegenerative illnesses have an impact on frontal lobe function (Butler and Chiong, 2019). It can be inferred that the disruption of structural and functional coupling found in the frontotemporal lobe is associated with cognitive impairment and that the burden of CSVD lesions corresponds with disruption in brain morphology and activity, which also promotes cognitive decline. Thus, the different trends of coupling values may be due to the different functional characteristics and damage degree of basal ganglia and frontotemporal lobe.

Furthermore, we found that the above key abnormal areas were more affected in the CSVD-s group than in the other two groups, while there was no apparent difference between the CSVD-m and control groups; this discordance was due to more severe pathological changes, correspondingly more varied MRI features, and more obvious disruption of brain topology in the CSVD-s group. It was proven that the classification of severe and mild groups based on the burden score is reliable, which is beneficial to the analysis of abnormal imaging indicators (e.g., structure the burden coupling) and the interpretation of pathophysiological processes in CSVD. In our previous study, we divided CSVD patients into groups according to whether they had CMBs and demonstrated alterations in ALFF and fALFF values, GMV and WMV in different brain regions in different groups (Feng et al., 2021; Li et al., 2022). To further explore CSVD, we need to conduct coupling analysis of ALFF with WMV and GMV to search for the deeper effects of diseases on the brain. To further explore CSVD, we need to conduct coupling analysis of ALFF with WMV and GMV to search for the deeper effects of diseases on the brain. Specifically, ALFF/VBM decoupling may be attributed to the asynchronism of disruptive brain activity and morphological structure or diminished correlations between functional interactions and underlying anatomy. The inconsistent direction of coupling values in various regions and the uneven change in ALFF and VBM indexes are likely related. The function of relevant regions is impacted by the structural changes caused by CSVD, and the primary functional abnormity may lead to secondary volume changes. One explanation for this phenomenon might be the compensatory effects of structure and function (Shang et al., 2019) in disease development. Namely, the intensive structure-function coupling in the frontotemporal region makes up for the reduced motor function and control arising from structure-function decoupling in the dorsal striatum. Therefore, ALFF/VBM decoupling can provide new explanations for the interaction of structure and function and the underlying pathophysiological mechanism of CSVD in patients.

Intriguingly, the mean ALFF/VBM values of all disrupted brain clusters achieved the significance level in ROC curve analysis. Among them, the mean ALFF/VBM value in the bilateral caudate nucleus performed the best, which indicated that structure–function coupling abnormalities in the caudate nucleus make a difference in discriminating CSVD patients from healthy controls. The caudate nucleus is a crucial component of brain executive functions, such as cognitive control, working memory and certain aspects of attention, such as the separation of attention (Aumont et al., 2019), and controls body movement. The caudate nucleus can be segmented into ventral and dorsal according to its connectivity and function, where the dorsal caudate nucleus is extraordinarily joined to the dorsolateral prefrontal cortex (DLPFC) and the latter is associated with executive function and working memory. The ventral caudate nucleus is more closely related to the limbic system and is related to emotional functions (Huang et al., 2017). CSVD-s patients are more prone to acquiring dementia, apathy, and vascular Parkinson’s disease as their disease worsens. In contrast, Parkinson’s disease and apathy are likely linked by structural abnormalities in the caudate nucleus (Gez et al., 2022). Therefore, the performance of ALFF-VBM coupling in the caudate nucleus was more impressive for distinguishing the CSVD-s group from the CSVD-m and control groups, suggesting that more attention should be given to changes in the function and/or structure of the caudate nucleus in CSVD patients in future studies.

Notably, CSVD burden is an important predictor of cognitive impairment in patients (Chen et al., 2019). Our recent studies demonstrated that alterations in brain structure (Sui et al., 2023) and function (Feng et al., 2021; Xin et al., 2022) were significantly correlated with cognitive dysfunction in CSVD patients. However, whether structure-function coupling plays a mediating role in the correlation between CSVD burden and cognitive parameters has not been explored. Our study may answer the above question. The main significant correlations observed were between the bilateral caudate nuclei and scores on the TMT, which are used to assess motor coordination executive functioning, and information processing speed (Wei et al., 2018). Our discoveries confirm the viewpoint that CSVD is the most universal mechanism leading to vascular cognitive impairment (Zanon Zotin et al., 2021) and that cognitive ability gradually declines with the gradual increase in the severity of CSVD burden. Cognitive impairment in the CSVD-s group with the most severe pathological changes may be closely related to abnormalities in the caudate nucleus, which can directly regulate motor control and indirectly affect executive function. Furthermore, our findings also showed that the correlation pattern between ALFF/VBM coupling and cognitive function was not consistent between CSVD-s and CSVD-m patients, especially in the inferior frontotemporal lobe. We speculate that the autoregulatory mechanisms of the brain may be able to respond, resulting in less pronounced ALFF/VBM decoupling and less consistent relevance between decoupling and cognitive function in CSVD-m patients. However, the regulating mechanism fails when the burden reaches a certain level. The inferior frontal gyrus is known to be closely connected with the dorsal caudate nucleus, forming a frontal-subcortical circuit mainly responsible for executive function (Raimo et al., 2019), and the hypometabolism and volume atrophy of the inferior frontotemporal gyrus are gradually aggravated with a worsening of CSVD burden. These results highlight the importance of investigating the correlation between structure-function coupling and cognitive function in patients with different CSVD burdens, suggesting that early screening, diagnosis, detection and treatment of CSVD in patients could prevent or delay cognitive decline.

Several limitations of this study need to be pointed out. Firstly, our study was cross-sectional, which limited us in terms of cause-and-effect analysis. A longitudinal design should be explored in future research to monitor individual neuroimaging and neuropsychological changes. Secondly, structural destruction of the lesion area could not be confirmed by histopathology due to our noninvasive analysis *in vitro*, which is another limitation. Thirdly, our prediction of dementia based on coupling abnormalities in different brain regions with different burdens of CSVD needs to be verified by further study of brain networks. Finally, the number of subjects in the CSVD-s group is relatively small compared to the other two groups. In the future, we will strengthen our recruitment efforts for more accurate and in-depth research.

## 5. Conclusion

In summary, significantly decreased ALFF/VBM coupling in the CSVD-s group was observed in the bilateral caudate nuclei, putamen and right IFG, and increased coupling was found in the right ITG, left MFG and SFGmed compared with the CSVD-m and control groups. In addition, ALFF/VBM decoupling in the caudate, IFG and ITG was significantly correlated with executive and attention functions in CSVD patients. Structure–function decoupling in the bilateral caudate nuclei is excellent at identifying patients with severe CSVD burden. These findings imply that ALFF/VBM coupling might be a powerful and innovative neuroimaging metric to track changes in CSVD burden and cognitive impairment and would be useful for the early diagnosis and prediction of CSVD progression. Early intervention plays a vital role in slowing cognitive decline and improving the quality of life of patients. In addition, this research offers new perspectives regarding the pathophysiological mechanisms involved in the clinical development of CSVD.

## Data availability statement

The original contributions presented in the study are included in the article/supplementary material, further inquiries can be directed to the corresponding authors.

## Ethics statement

The studies involving human participants were reviewed and approved by the Shandong Provincial Hospital Affiliated with Shandong First Medical University’s Institutional review board. The patients/participants provided their written informed consent to participate in this study.

## Author contributions

XZ and CL wrote the main manuscript text. NW, YW, YG, CS, MF, and HX prepared the clinical data and imaging data. HW and LG revised the main manuscript text. All authors reviewed the manuscript and contributed to the article and approved the submitted version.

## Footnotes

1
www.mocatest.org

2
http://rfmri.org/dpabi

3
http://www.fil.ion.ucl.ac.uk/spm

4
https://www.medcalc.org
